# The Importance of Bottlenecks in Protein Networks: Correlation with Gene Essentiality and Expression Dynamics

**DOI:** 10.1371/journal.pcbi.0030059

**Published:** 2007-04-20

**Authors:** Haiyuan Yu, Philip M Kim, Emmett Sprecher, Valery Trifonov, Mark Gerstein

**Affiliations:** 1 Department of Molecular Biophysics and Biochemistry, Yale University, New Haven, Connecticut, United States of America; 2 Department of Genetics, Harvard Medical School, Boston, Massachusetts, United States of America; 3 Department of Cancer Biology, Dana-Farber Cancer Institute, Boston, Massachusetts, United States of America; 4 Program in Computational Biology and Bioinformatics, Yale University, New Haven, Connecticut, United States of America; 5 Department of Computer Science, Yale University, New Haven, Connecticut, United States of America; Weill Medical College of Cornell University, United States of America

## Abstract

It has been a long-standing goal in systems biology to find relations between the topological properties and functional features of protein networks. However, most of the focus in network studies has been on highly connected proteins (“hubs”). As a complementary notion, it is possible to define bottlenecks as proteins with a high betweenness centrality (i.e., network nodes that have many “shortest paths” going through them, analogous to major bridges and tunnels on a highway map). Bottlenecks are, in fact, key connector proteins with surprising functional and dynamic properties. In particular, they are more likely to be essential proteins. In fact, in regulatory and other directed networks, betweenness (i.e., “bottleneck-ness”) is a much more significant indicator of essentiality than degree (i.e., “hub-ness”). Furthermore, bottlenecks correspond to the dynamic components of the interaction network—they are significantly less well coexpressed with their neighbors than nonbottlenecks, implying that expression dynamics is wired into the network topology.

## Author Summary

A network is a graph consisting of a number of nodes with edges connecting them. Recently, network models have been widely applied to biological systems. Here, we are mainly interested in two types of biological networks: the interaction network, where nodes are proteins and edges connect interacting partners; and the regulatory network, where nodes are proteins and edges connect transcription factors and their targets. Betweenness is one of the most important topological properties of a network. It measures the number of shortest paths going through a certain node. Therefore, nodes with the highest betweenness control most of the information flow in the network, representing the critical points of the network. We thus call these nodes the “bottlenecks” of the network. Here, we focus on bottlenecks in protein networks. We find that, in the regulatory network, where there is a clear concept of information flow, protein bottlenecks indeed have a much higher tendency to be essential genes. In this type of network, betweenness is a good predictor of essentiality. Biological researchers can therefore use the betweenness as one more feature to choose potential targets for detailed analysis.

## Introduction

Protein networks are a topic of great current interest, particularly after a growing number of large-scale protein networks have been determined [1–6]. Protein–protein interaction networks and regulatory networks are the key representatives for biological networks with undirected and directed edges [7–12]. Previous topological studies were mainly focused on analyzing degree distributions and finding motifs within these networks [7–9,11,13,14]. All of these networks are scale-free with power-law degree distributions, and hubs (proteins with high degrees) in the network represent the most vulnerable points [7–9,14].

Recently, another topological feature of the network has received attention—betweenness, which measures the total number of nonredundant shortest paths going through a certain node or edge [15,16]. Betweenness was originally introduced to measure the centrality of the nodes in networks [15]. By definition, most of the shortest paths in a network go through the nodes with high betweenness. Therefore, these nodes become the central points controlling the communication among other nodes in the network. More recently, Girvan and Newman proposed that the edges with high betweenness are the ones that are “between” highly interconnected subgraph clusters (i.e., “community structures”); therefore, removing these edges could partition a network [16]. Furthermore, Dunn et al. found that protein clusters within interaction networks defined by this edge betweenness method tend to share similar functions [17].

**Figure 1:**
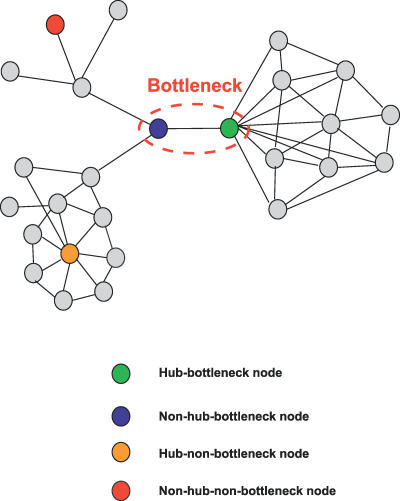
Schematic Showing a Bottleneck and the Four Categories of Nodes in a Network Four nodes with different colors represent examples of the four categories defined by degree and betweenness. Please note that every node in the network belongs to one of the four categories. However, in this schematic, we only point out the categories of the four example nodes.

Here, we revisited the original meaning of betweenness as a measure of the centrality of the nodes in a network. If we think of protein networks (in particular, regulatory networks) in analogy to a transportation network, proteins with high betweenness are similar to heavily used intersections, such as those leading to major highways or bridges (see Figure 1). If these major intersections were blocked, there would be huge traffic jams, causing the whole transportation system to fail. Therefore, we called these high-betweenness proteins bottlenecks, and hypothesized that these bottlenecks, just like hubs, represent important points in biological networks as well. For simplicity, we defined protein bottlenecks as the proteins with the highest betweenness; hubs, as the proteins with the highest degree (see Methods).

In fact, previous studies have shown that protein bottlenecks are indeed more likely to be essential [18,19]. This holds true in three different eukaryotic protein-interaction networks: yeast, worm, and fly [19]. However, Goh and his colleagues also found that, in these interaction networks, the betweenness of a node is correlated to its degree [20]. Therefore, it is not clear whether protein bottlenecks are important because they have high betweenness or because they also tend to be hubs.

Moreover, Han et al. found that there are two categories of protein hubs within the yeast protein-interaction network: “party hubs” interact with most of their partners simultaneously, whereas “date hubs” bind theirs asynchronously [21]. Because protein bottlenecks in the interaction network connect different functional clusters—as mentioned above in [17]—it is conceivable that bottlenecks with high degrees should have a higher tendency to be date hubs.

Here, we analyzed the biological significance of betweenness in terms of protein functions, expression correlation, and its relationships with protein hubs.

## Results

### Bottlenecks Tend To Be Essential

**Figure 2:**
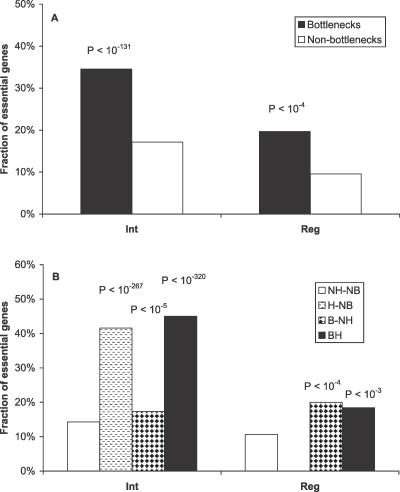
Comparison of Essentiality among Various Categories of Proteins within Interaction and Regulatory Networks (A) Bottlenecks tend to be essential genes in both interaction and regulatory networks. *p-*Values measure the statistical significance of the different essentialities between bottlenecks and nonbottlenecks. (B) Essentiality of different categories of proteins. NH-NB, nonhub–nonbottlenecks; H-NB, hub–nonbottlenecks; B-NH, nonhub–bottlenecks; BH, hub–bottlenecks. *p-*Values measure the statistical significance of the different essentialities between different categories of proteins against nonhub–nonbottlenecks using cumulative binomial distributions (see Methods).

Because bottlenecks are key connectors in protein networks, we hypothesized that these proteins would represent important points in networks. Therefore, we first examined the essentiality of bottlenecks in different networks in yeast (see Methods). We found that bottlenecks in both regulatory and interaction networks indeed tend to be essential proteins with high significance (see Figure 2A), in agreement with previous studies [18,19].

### Bottlenecks Dictate the Essentiality of Networks with Directed Edges

As discussed above, previous studies have shown that, in biological networks, hubs tend to be essential [7,9], and betweenness of a node is correlated with its degree [20]. We found that degree and betweenness are indeed highly correlated quantities in the networks we analyzed (Pearson correlation coefficient of 0.49, *p* < 10^−15^ for the interaction network; Pearson correlation coefficient of 0.67, *p* < 10^−15^ for the regulatory network; *p*-values measure the significance of the Pearson correlation coefficient scores according to *t* distributions; i.e., many bottlenecks also tend to be hubs). Therefore, we further investigate which one of these two quantities is a better predictor of protein essentiality in both regulatory and interaction networks.

To disentangle the effects of betweenness and degree, we divided all proteins in a certain network into four categories: (1) nonhub–nonbottlenecks; (2) hub–nonbottlenecks; (3) nonhub–bottlenecks; and (4) hub–bottlenecks (see Figure 1). Even though the two quantities are highly correlated, the number of hub–nonbottlenecks and nonhub–bottlenecks is enough for reliable statistics (see Table S1). This is in agreement with the previous observation by Huang and his colleagues, who found that proteins with high betweenness but low degree (i.e., nonhub–bottlenecks) are abundant in the yeast protein interaction network [18].

As we discussed above, numerous previous studies have shown that the degree of a protein determines its essentiality in scale-free networks (i.e., proteins with higher degrees are more likely to be essential) [7,9]. Both interaction and regulatory networks have been shown to be scale-free networks [7,9,22]. Here, we observed that bottlenecks (both nonhub–bottlenecks and hub–bottlenecks) have a strong tendency to be products of essential genes, whereas hub–nonbottlenecks are surprisingly not essential. Thus, we determined that it is the betweenness that is a stronger determinant of the essentiality of a protein in the regulatory network, not the degree (Figure 2B).

**Figure 3:**
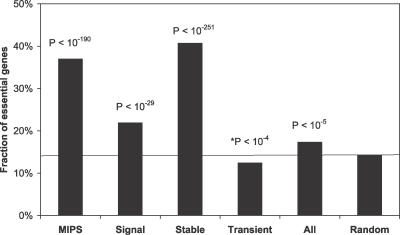
Essentialities of Different Categories of Nonhub–Bottlenecks in the Interaction Network To find all proteins participating in signaling transduction pathways (i.e., the bar “Signal”), we manually went through all available pathways in KEGG and collected all proteins in them. Since this is a very small dataset, we further included all proteins, more than half of whose interacting partners are involved in signaling transduction pathways in KEGG. This inclusion is reasonable because the general belief is that interacting proteins share the same function. The fraction of essential genes among nonhub–nonbottlenecks is used as the random expectation, which is also indicated by the horizontal line. *p-*Values measure the statistical significance of the different essentialities of different categories of nonhub–bottlenecks relative to the random expectation. The bar “MIPS” refers to nonhub–bottlenecks involved in the complexes defined by the MIPS complex catalog. The bar “Permanent” refers to nonhub–bottlenecks involved in permanent interactions. The bar “Transient” refers to nonhub–bottlenecks only involved in transient interactions. The bar “All” refers to all nonhub–bottlenecks. *p-Value above the bar “Transient” measures the statistical significance of transient nonhub–bottlenecks being less essential than random.

In contrast to regulatory networks, the interaction network is undirected with no obvious information flow. Furthermore, nonneighboring pairs in the interaction network have no noticeable relationships, as they are neither coregulated nor coexpressed [23]. Therefore, it is reasonable to assume that in interaction networks, hubs are more important than bottlenecks. Our calculations confirmed this hypothesis (see Figure 2B): although nonhub–bottlenecks are significantly more likely to be essential than nonhub–nonbottlenecks (*p* < 10^−5^; see Methods for the calculation of *p-*values in Figures 2 and 3), the difference is not nearly as substantial as that between hub–nonbottlenecks and nonhub–nonbottlenecks (*p* < 10^−267^). Similar results were also found in different interaction networks (see Figure S4). The difference between nonhub–bottlenecks (low essentiality) and hub–nonbottlenecks (much higher essentiality) confirms that degree is a much better predictor of essentiality in the interaction network.

Signal transduction pathways are a special case of protein–protein interactions [24]. There are well-defined information flows in these pathways. Nonhub–bottlenecks participating in signaling transduction pathways clearly are more likely to be products of essential genes (see Figure 3).

### Bottlenecks within Permanent Undirected Interactions Are Also Important

Besides directionality, another important but often overlooked aspect of interaction networks is that there are two major classes of interactions: permanent and transient [25,26]. Within permanent interactions, bottlenecks are connectors holding different, functionally important complexes together. However, within transient interactions, bottlenecks merely interact with different complexes at different times. In this sense, “transient” bottlenecks are not really bottlenecks. They are classified as “bottlenecks” by our algorithm simply because of the fact that current interaction networks are a collection of individual networks under various conditions. As a result, the function of these transient bottlenecks is likely to be not as important as that of permanent ones. Therefore, we hypothesized that bottlenecks would be more likely to be essential in permanent rather than in transient interactions.

We tested our hypothesis in the yeast interaction network. Defining permanent interactions as those participating in protein complexes, we analyzed all complexes from the Munich Information Center for Protein Sequences (MIPS) complex catalog [27]. (Previous studies have shown that most of the MIPS complexes are stable, permanent complexes. However, there are 52 proteins in this catalog without direct evidence of stable interactions with others [26]. We removed these proteins from our analysis.) Because the catalog is far from complete, we also considered all interactions forming a clique (a complete subgraph) of size 5 or bigger as permanent, because protein complexes are often considered as cliques in interaction networks [28,29]. Any interaction not participating in a clique of size 3 or bigger was considered transient. Our calculations confirmed our hypothesis: nonhub–bottlenecks within permanent interactions tend to be essential, while those within transient ones do not (see Figure 3).

For completeness, we also analyzed hubs and bottlenecks in other kinds of protein networks. Specifically, we analyzed the topology of three very different kinds of protein networks, namely the metabolic network (where links connect enzymes that share a metabolite) [23], the genetic network (where links connect proteins that have genetic interactions) [30], and the phosphorylation network (where links connect a kinase with its substrates) [31]. The edges in the phosphorylation and metabolic networks are directed, whereas those in the genetic network are undirected (see Table S2 and http://www.gersteinlab.org/proj/bottleneck).

### Bottlenecks Constitute the Dynamic Components of Networks

The correspondence of protein interaction bottlenecks to connectors both in complexes and in pathways leads us to investigate their dynamic properties. To this end, we examined their coexpression with their neighbors. It has been previously observed that interacting protein pairs are more likely to coexpress than noninteracting protein pairs [32]. Likewise, protein complex members have been shown to be highly coexpressed [25]. Given this information, we hypothesized that bottlenecks would tend to have a below-average expression correlation with their neighbors, since they tend to represent proteins that connect different complexes or pathways. Indeed, in all the datasets examined, we find that bottlenecks have a much lower average expression correlation with their neighbors than other nodes.

**Figure 4:**
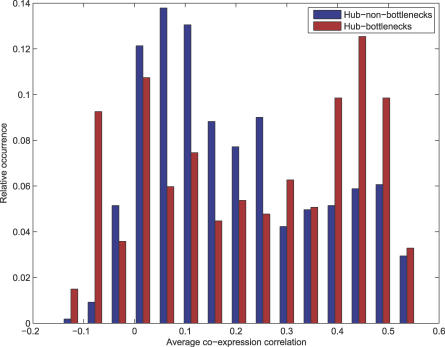
Expression Dynamics of Hub–Nonbottlenecks and Hub–Bottlenecks Histograms of the average correlation coefficient of the expression profile of any given gene with its direct interaction partners. Expression dynamics for all four categories of nodes are shown in Figure S7.

Surprisingly, the difference is much more pronounced when focusing on hubs only (i.e., the difference is more significant between hub–nonbottlenecks and hub–bottlenecks than between nonhub–nonbottlenecks and nonhub–bottlenecks). The majority of hub–nonbottlenecks are relatively well coexpressed with their neighbors, whereas most hub–bottlenecks are not very well coexpressed (see Figure 4). What appears especially striking is that bottlenecks always have low coexpression with their neighbors, whereas hubs can have a relatively high average coexpression with their neighbors, but only if they are nonbottlenecks. We find that while nonbottlenecks simply follow the same distribution as the rest of the datasets, the nonhub–bottlenecks tend to have a lower expression correlation.

Central complex members have a low betweenness and are hub–nonbottlenecks. Because of the high connectivity inside these complexes, paths can go through them and all their neighbors. On the other hand, hub–bottlenecks tend to correspond to highly central proteins that connect several complexes or are peripheral members of central complexes. The fact that they have a high betweenness suggests that these proteins are not, however, simply members of large protein complexes (which is true for nonbottleneck–hubs), but are those members that connect the complex to the rest of the graph; in a sense, real connectivity bottlenecks. While hub–nonbottlenecks mainly consist of structural proteins, hub–bottlenecks are more likely to be part of signal transduction pathways (see Table S3). Furthermore, hub–bottlenecks are (by construction) the most efficient in disrupting the network upon hub removal (see Figure S3). This relates nicely to the date/party-hub concept by Han et al. [21]: hub–bottlenecks tend to be date-hubs, whereas hub–nonbottlenecks tend to be party-hubs.

Nonhub–bottlenecks generally coexpress less well with their neighbors than nonhub–nonbottlenecks, in line with the observation that betweenness is a good predictor of average correlation with neighbors. Nonhub–bottlenecks also rarely are complex members and are in large part made up of regulatory proteins and signal transduction machinery.

## Discussion

In this study, we find surprising links between network topology and both protein phenotype and expression dynamics. In analogy to the well-known network hubs, we examined the properties of so-called network bottlenecks in yeast. A first surprising finding is the distinction between interaction and regulatory networks in the relative importance of bottlenecks to hubs. While in most topological features, regulatory networks have been thought of as similar to interaction networks, we clearly see a distinction between those two network types that leads to a direct biological interpretation. Regulatory networks have directed edges; there is an implicit information flow within the network, which makes it more similar to the transportation system. A transcription factor (TF) can regulate many target genes indirectly through other TFs. Deletion of TF bottlenecks thus leads to the disruption of a large number of direct and indirect regulations between TFs and their targets and is lethal to the cell. For example, Swi1p is a nonhub–bottleneck TF required for sporulation and other cellular processes [33]. Swi1p is not a hub with only 23 targets. But, it is controlled by four TFs, and also regulates four others [34,35]. Because of this unique topological position, approximately 10,000 shortest paths between TFs and their targets within the whole regulatory network run through this gene, making it an important bottleneck. As a result, Swi1p is essential for viability in yeast [36].

On the other hand, protein–protein interaction networks have undirected edges; there is no obvious information flow within the network. Therefore, some people may even argue that in these interaction networks, betweenness, as well as the definition of bottlenecks, is more of a topological conceptualization from an abstract graph-theory point of view without clear biological meanings. Our calculations confirm accordingly that degree is a much better predictor of essentiality in interaction networks. More interesting, in some subnetworks within interaction networks where betweenness does have biological implications (e.g., subnetworks involved in signaling transduction or permanent interactions), protein bottlenecks indeed have a higher tendency to be essential. All of these correlations between topological measurements (namely, degree and betweenness) that we discovered here are quite intuitive if we carefully examine the topological meanings of these measurements and the biological interpretation of these networks.

**Figure 5:**
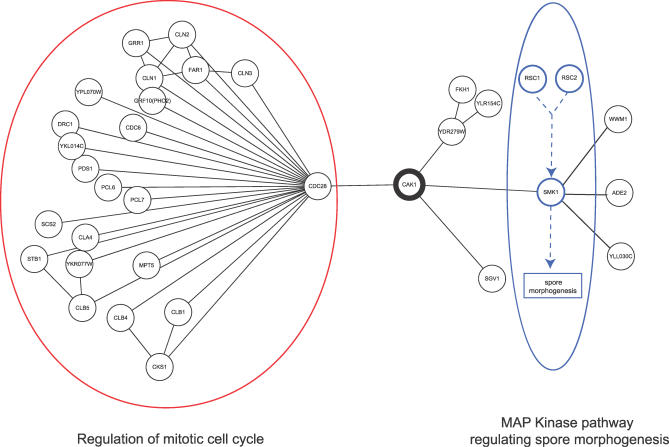
A Biological Example of a Nonhub–Bottleneck in the Interaction Network Cak1p is a cyclin-dependent kinase-activating kinase involved in two key signaling-transduction pathways: cell cycle and sporulation.

Moreover, our approach of focusing on nonhub–bottlenecks is useful for finding proteins that mediate different processes and are involved in cross-talk. An example is Cak1p (see Figure 5), which is a cyclin-dependent kinase-activating kinase involved in two key signaling-transduction pathways. It activates Cdc28p, an important regulator of the cell cycle. Cak1p also induces Smk1p, a mitogen-activated protein kinase involved in sporulation [37,38]. Besides these two proteins, Cak1p only has two other interaction partners (YDR279W and Sgv1p), making its total degree 4. Therefore, it is not a hub in the interaction network. However, since it coordinates two major signaling-transduction pathways, it is an important nonhub–bottleneck in the network with a high betweenness of 16,832.95 paths. Finally, due to its unique topological position in the network, *CAK1* is an essential gene in the cell. More interestingly, it is also a close homolog of the human cancer gene *CDK6* (BLAST E-value < 10^−10^). This example shows that bottlenecks potentially could be applied in various medical and pharmaceutical contexts to identify key proteins.

Generally, the protein interaction network and gene expression data are generally viewed as independent. While there were several studies addressing correlations among them, they focused largely on local properties [32]. Likewise, while many studies addressed relations between the interaction network and protein function, they only make use of local network features, such as distance [39–42]. Here, we show that both coexpression and essentiality are highly correlated with a global network feature, betweenness. This finding lets us view the interaction network in a different light—some dynamic information is wired into the topology. This finding reinforces the “date-hub” and “party-hub” concept suggested by Han et al. [21]. It appears that the property of betweenness separates the bimodal distribution of average coexpression in hubs. Thus, the so-called date-hubs correspond mostly to hubs with high betweenness (hub-bottlenecks), while the “party-hubs” correspond mostly to hubs with low betweenness (hub–nonbottlenecks). This finding, however, implies relationships between dynamics and topological properties in the interaction network that were hitherto unknown.

It is possible to argue that there is a certain level of noise in our interaction dataset, even though it is a highly reliable set [23,43]. To demonstrate that our results are not due to some specific noise in our dataset, we repeated all calculations on other high-quality interaction datasets (namely, the filtered yeast interactome [FYI] [21] and the DIP core [44]) as well. These different datasets all exhibit similar results (see Figures S1 and S4A).

Finally, a principal contribution of this paper is the consistent calculation of betweenness on directed and undirected graphs. We also performed our calculations on all currently available yeast protein networks with directed and undirected edges. Most of these networks are much smaller than the interaction and regulatory networks. So, calculating robust statistics is not currently possible, but we believe that as these other networks grow in size in the future, betweenness will prove to be a useful quantity for many protein networks, particularly those with directed edges. As described in Methods, we plan to regularly update our website (http://www.gersteinlab.org/proj/bottleneck) with betweenness calculations as these networks grow.

In summary, we present an integrated analysis of two complementary topological network properties across different network types. This combined approach uncovers previously unknown connections between network topology, protein essentiality, and expression dynamics. We believe that integrated approaches like the one presented here will be of paramount importance in future predictive models.

## Materials and Methods

### Data sources.

Interaction data was gathered from a number of different published high-throughput datasets and published databases [2–5,27,34,45,46]. Independent genomic features and Bayesian integration were used to eliminate noise from the dataset [23,43]. Different datasets (e.g., the FYI [Vidal et al.] [21] or the DIP core [Eisenberg et al.] [44]) exhibit the same behavior (see Figures S1 and S4A). To avoid biases from large complexes (i.e., the ribosome and the proteasome), we repeated our calculations after removing both these complexes (see Figures S2 and S4B). The regulatory network was created by combining five different datasets [1,2,22,34,35,47]. We excluded DNA-binding enzymes (e.g., PolIII) from the regulatory network. The essential genes in yeast genome were determined experimentally through a PCR-based gene-deletion method [36]. The metabolic network was taken from the Kyoto Encyclopedia of Genes and Genomes (KEGG) [48] and all proteins that share a metabolite were considered linked. The genetic network data was downloaded from the GRID [49] and consists of several large-scale screens of genetic interactions [30,50]. Expression data was taken from the Rosetta compendium expression dataset [51]. All datasets and the calculated betweenness of each protein node within these networks are available at http://www.gersteinlab.org/proj/bottleneck. Because most of these networks are far from complete, we will update the networks and, more important, the associated betweenness of each node as they grow in size in the future.

### Definition of hubs and bottlenecks.

We defined hubs as all proteins that are in the top 20% of the degree distribution (i.e., proteins that have the 20% highest number of neighbors). Accordingly, we defined bottlenecks as the proteins that are in the top 20% in terms of betweenness. Varying this cutoff from 10% to 40% had no significant impact on our results (see Figures S5–S7).

### Algorithm to calculate betweenness.

To calculate node betweenness within networks [16,52], we used an improved version of the algorithm developed by Newman and Girvan. (1) Initialize the betweenness of every vertex *v* in the network *B_v_* = 0. (2) Starting from a vertex *i*, a breadth-first tree is built with *i* on the top and those that are farthest from *i* at the bottom [53]. Each node is put at a certain level of the tree based on its shortest distance from *i*. (3) A variable *p_i_* = 1 is assigned to *i*. As we are building the tree, for every vertex *j*,

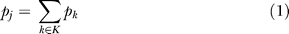
where *K* is the set of nodes that directly connect to *j* and are at the immediate proceeding level (i.e., predecessors of *j*). (4) Another variable *b_j_*, with an initial value of 1, is also assigned to every vertex *j* in the tree. (5) Starting from a bottom vertex *j*, the value of *b_j_* is added to the corresponding variable of the predecessor of *j*. If *j* has more than one predecessor, each predecessor *k* gets the value of:

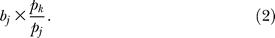
Therefore:

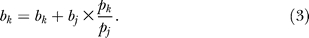
(6) Perform step 5 for every vertex in the tree. (7) For every vertex *j* in the tree, *B_j_* = *B_j_* + *b_j_*. (8) Repeat steps 2–7 for every vertex in the network.

Qualitatively, proteins with high betweenness are considered as bottlenecks. To facilitate our calculations and discussion, however, we quantitatively defined bottlenecks as the top 20% proteins with the highest betweenness values, in agreement with the conventional cutoff for protein hubs [9]. Please note that for networks with directed edges, the directionality of the edges have to be taken into consideration.

### *p-*Values by cumulative binomial distribution.

*p*-Values in Figures 2 and 3 measure whether the difference is significant between the testing and control groups. They are calculated using the cumulative binomial distribution:

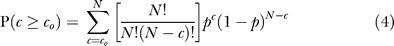
where *N* is the total number of genes in the data; *c_o_* is the number of observed genes with a specific property (e.g., essentiality) in the testing group; and *p* is the probability of finding a gene with the same property in the control group. In this manner, we are testing whether genes with a specific property are overrepresented compared with the control group. If they are underrepresented, then P(*c* < *c_o_*) = 1 − P(*c* ≥ *c_o_*).

## Supporting Information

Figure S1 The Average Expression Correlation for Hub–Bottlenecks, Nonhub–Bottlenecks, Hub–Nonbottlenecks, and Nonhub–Nonbottlenecks for the FYI The trend is similar to the one seen in Figure 4, with hub–bottlenecks always having low correlations and nonhub–bottlenecks mostly having higher expression correlations. The fact that the signal is weaker is likely due to the small size of the FYI dataset.
(12 KB PDF)

Figure S2 The Average Expression Correlation for Hub–Bottlenecks, Nonhub–Bottlenecks, Hub–Nonbottlenecks, and Nonhub–Nonbottlenecks after Removal of Large Protein Complexes (Ribosomes and Proteasome) As can be seen, the trend for nonhub–bottlenecks to have high average coexpression is still clearly discernible. It can be seen more clearly that there is a bimodal distribution of nodes with low and high coexpression, with the nonhub–bottlenecks being highly enriched for high coexpression values.
(12 KB PDF)

Figure S3 Bottlenecks (Including Nonhub–Bottlenecks), but Not Hub–Nonbottlenecks, Are Crucial Nodes for Topological Integrity of the Network As can be seen clearly, the removal of bottlenecks (or nonhub–bottlenecks) leads to the breakdown of network topology much quicker than the removal of hubs or even hub–nonbottlenecks.
(14 KB PDF)

Figure S4 Fraction of Essential Genes among the Four Types of Nodes (i.e., Hub–Bottlenecks, Nonhub–Bottlenecks, Hub–Nonbottlenecks, and Nonhub–Nonbottlenecks) for Different Interaction Networks (A) FYI interaction network used by Han et al. [21].
(B) Interaction networks without large complexes (ribosomes and proteasomes).
BH, hub–bottlenecks; NH–B, nonhub–bottlenecks; H–NB: hub–nonbottlenecks; and NH–NB, nonhub–nonbottlenecks.
(38 KB PDF)

Figure S5 Fraction of Essential Genes among the Four Types of Nodes (i.e., Hub–Bottlenecks, Nonhub–Bottlenecks, Hub–Nonbottlenecks, and Nonhub–Nonbottlenecks) by Using Different Cutoffs for Hubs and Bottlenecks (A) Interaction network.
(B) Regulatory network.
BH, hub–bottlenecks; NH–B, nonhub–bottlenecks; H–NB: hub–nonbottlenecks; and NH–NB, nonhub–nonbottlenecks.
(81 KB PDF)

Figure S6 The Average Expression Correlation for Nonhub–Bottlenecks and Hub–Nonbottlenecks by Using Different Cutoffs for Hubs and Bottlenecks (A) The cutoff is 1%.
(B) The cutoff is 5%.
(C) The cutoff is 10%.
(D) The cutoff is 20%.
(25 KB PDF)

Figure S7 The Average Expression Correlation for Hub–Bottlenecks, Nonhub–Bottlenecks, Hub–Nonbottlenecks, and Nonhub–Nonbottlenecks by Using Different Cutoffs for Hubs and Bottlenecks (A) The cutoff is 1%.
(B) The cutoff is 5%.
(C) The cutoff is 10%.
(D) The cutoff is 20%.
(37 KB PDF)

Table S1 Number of Proteins in Each of the Four Categories in Both Interaction and Regulatory Networks (44 KB DOC)

Table S2 Essentiality of Different Categories of Proteins in Phosphorylation, Metabolic, and Genetic Networks (28 KB DOC)

Table S3 Occurrence of Different Biological Process Annotations from the Gene Ontology Annotation Scheme in the Four Different Topological Categories (54 KB DOC)

## Abbreviations

FYI: filtered yeast interactome
MIPS: Munich Information Center for Protein Sequences
TF: transcription factor
