# Supplementary material for: The Importance of Bottlenecks in Protein Networks: Correlation with Gene Essentiality and Expression Dynamics
Source: PLoS Comput Biol. 2007 Apr 20;3(4):e59. doi: 10.1371/journal.pcbi.0030059 (PMC1853125; doi:10.1371/journal.pcbi.0030059)
Supplement: Supplementary file 1 [file pcbi.0030059.sg001.pdf]

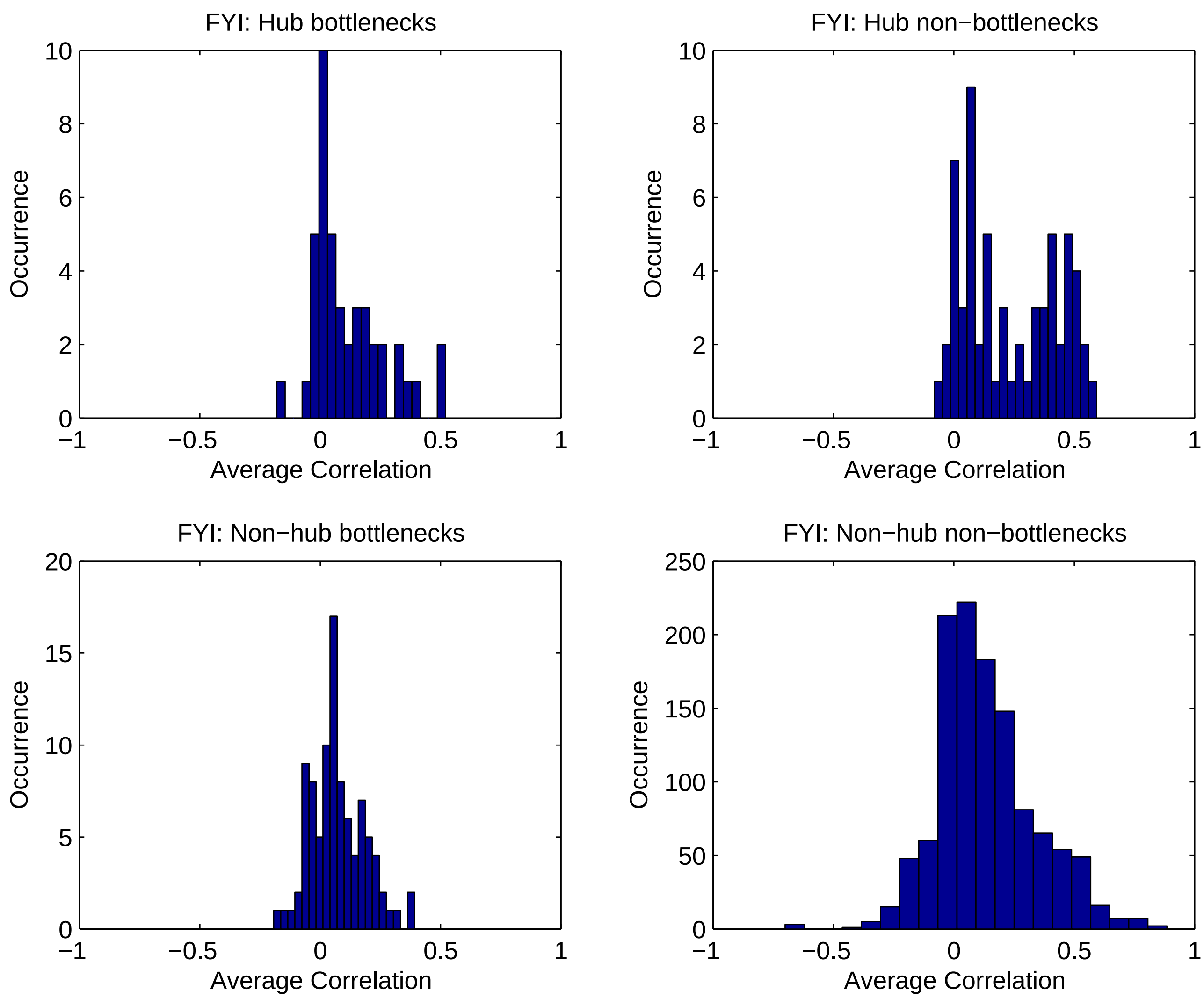
FYI: Hub bottlenecks
FYI: Hub non−bottlenecks
FYI: Non−hub bottlenecks
FYI: Non−hub non−bottlenecks
Occurrence
Average Correlation
−1
−0.5
0
0.5
1
10
8
6
4
2
0
20
15
5
250
200
150
100
50

SupFig1
