# Supplementary material for: The Importance of Bottlenecks in Protein Networks: Correlation with Gene Essentiality and Expression Dynamics
Source: PLoS Comput Biol. 2007 Apr 20;3(4):e59. doi: 10.1371/journal.pcbi.0030059 (PMC1853125; doi:10.1371/journal.pcbi.0030059)
Supplement: Supplementary file 2 [file pcbi.0030059.sg002.pdf]

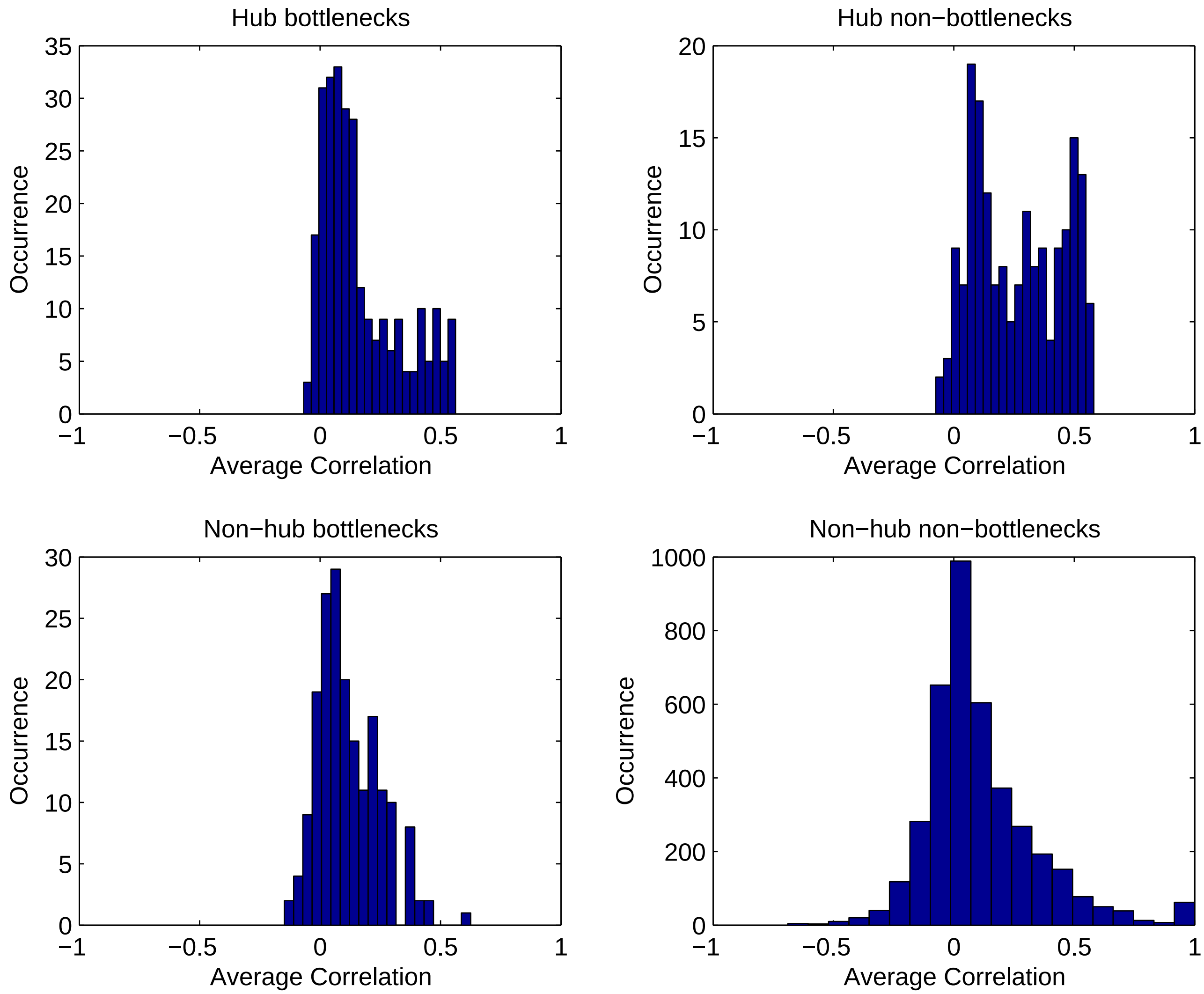

Hub bottlenecks
Occurrence
Average Correlation
35
30
25
20
15
10
5
0
−1
−0.5
0
0.5
1
Hub non−bottlenecks
Occurrence
Average Correlation
20
15
10
5
0
Non−hub bottlenecks
Occurrence
Average Correlation
30
25
20
15
10
5
0
Non−hub non−bottlenecks
Occurrence
Average Correlation
1000
800
600
400
200
0

SupFig2
