# Supplementary material for: The Importance of Bottlenecks in Protein Networks: Correlation with Gene Essentiality and Expression Dynamics
Source: PLoS Comput Biol. 2007 Apr 20;3(4):e59. doi: 10.1371/journal.pcbi.0030059 (PMC1853125; doi:10.1371/journal.pcbi.0030059)
Supplement: Supplementary file 3 [file pcbi.0030059.sg003.pdf]

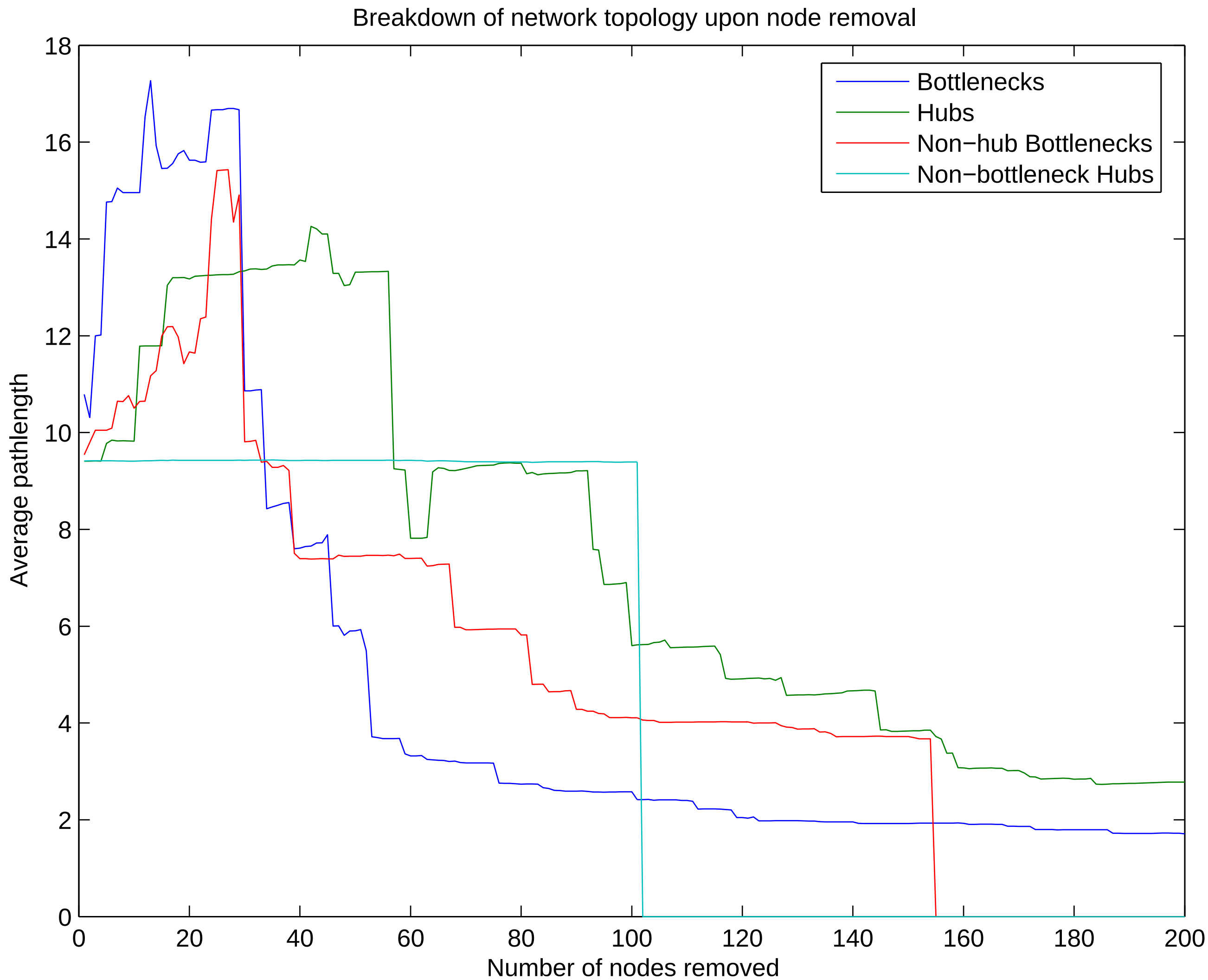

Breakdown of network topology upon node removal
Average pathlength
Number of nodes removed
0
2
4
6
8
10
12
14
16
18
0
20
40
60
80
100
120
140
160
180
200
Bottlenecks
Hubs
Non−hub Bottlenecks
Non−bottleneck Hubs

SupFig3
