# Supplementary material for: The Importance of Bottlenecks in Protein Networks: Correlation with Gene Essentiality and Expression Dynamics
Source: PLoS Comput Biol. 2007 Apr 20;3(4):e59. doi: 10.1371/journal.pcbi.0030059 (PMC1853125; doi:10.1371/journal.pcbi.0030059)
Supplement: Supplementary file 4 [file pcbi.0030059.sg004.pdf]

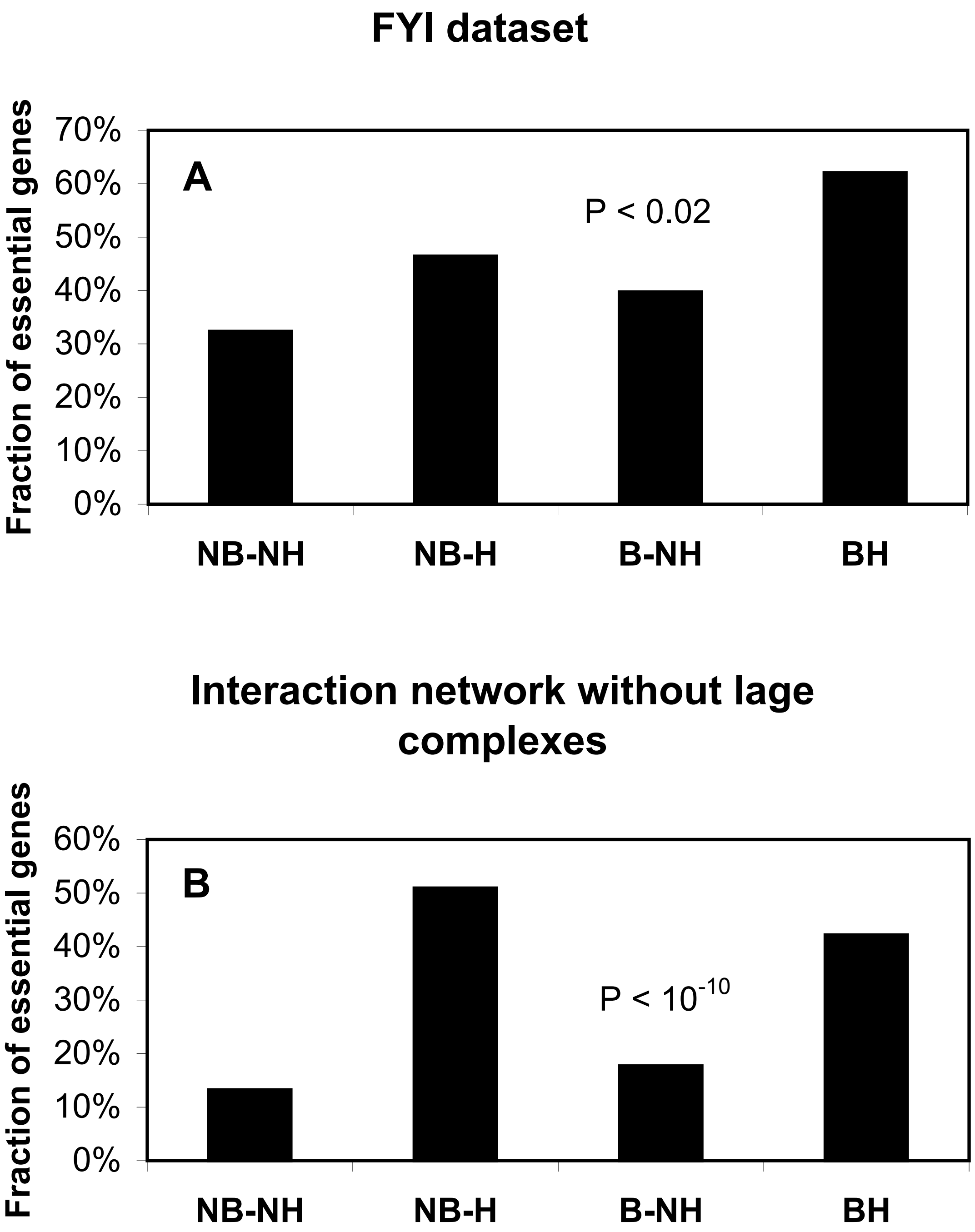
FYI dataset
Fraction of essential genes
70%
60%
50%
40%
30%
20%
10%
0%
A
P < 0.02
NB-NH
NB-H
B-NH
BH
Interaction network without lage complexes
Fraction of essential genes
60%
50%
40%
30%
20%
10%
0%
B
P < 10-10
NB-NH
NB-H
B-NH
BH

SupFig4
