# Supplementary material for: The Importance of Bottlenecks in Protein Networks: Correlation with Gene Essentiality and Expression Dynamics
Source: PLoS Comput Biol. 2007 Apr 20;3(4):e59. doi: 10.1371/journal.pcbi.0030059 (PMC1853125; doi:10.1371/journal.pcbi.0030059)
Supplement: Supplementary file 5 [file pcbi.0030059.sg005.pdf]

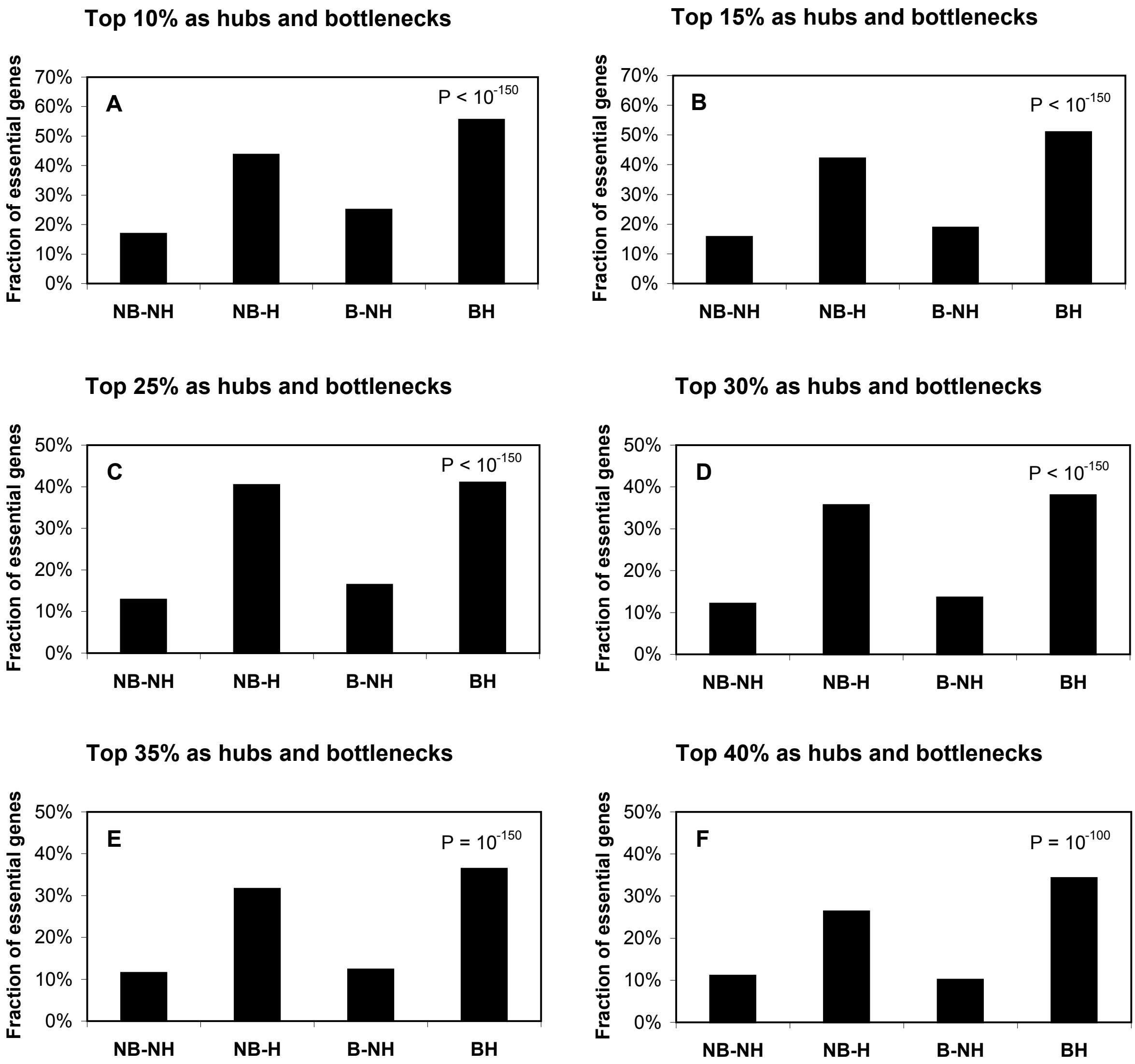
Top 10% as hubs and bottlenecks
Fraction of essential genes
70%
60%
50%
40%
30%
20%
10%
0%
A
P < 10-150
NB-NH
NB-H
B-NH
BH
Top 15% as hubs and bottlenecks
Fraction of essential genes
70%
60%
50%
40%
30%
20%
10%
0%
B
P < 10-150
NB-NH
NB-H
B-NH
BH
Top 25% as hubs and bottlenecks
Fraction of essential genes
50%
40%
30%
20%
10%
0%
C
P < 10-150
NB-NH
NB-H
B-NH
BH
Top 30% as hubs and bottlenecks
Fraction of essential genes
50%
40%
30%
20%
10%
0%
D
P < 10-150
NB-NH
NB-H
B-NH
BH
Top 35% as hubs and bottlenecks
Fraction of essential genes
50%
40%
30%
20%
10%
0%
E
P = 10-150
NB-NH
NB-H
B-NH
BH
Top 40% as hubs and bottlenecks
Fraction of essential genes
50%
40%
30%
20%
10%
0%
F
P = 10-100
NB-NH
NB-H
B-NH
BH

SupFig5A

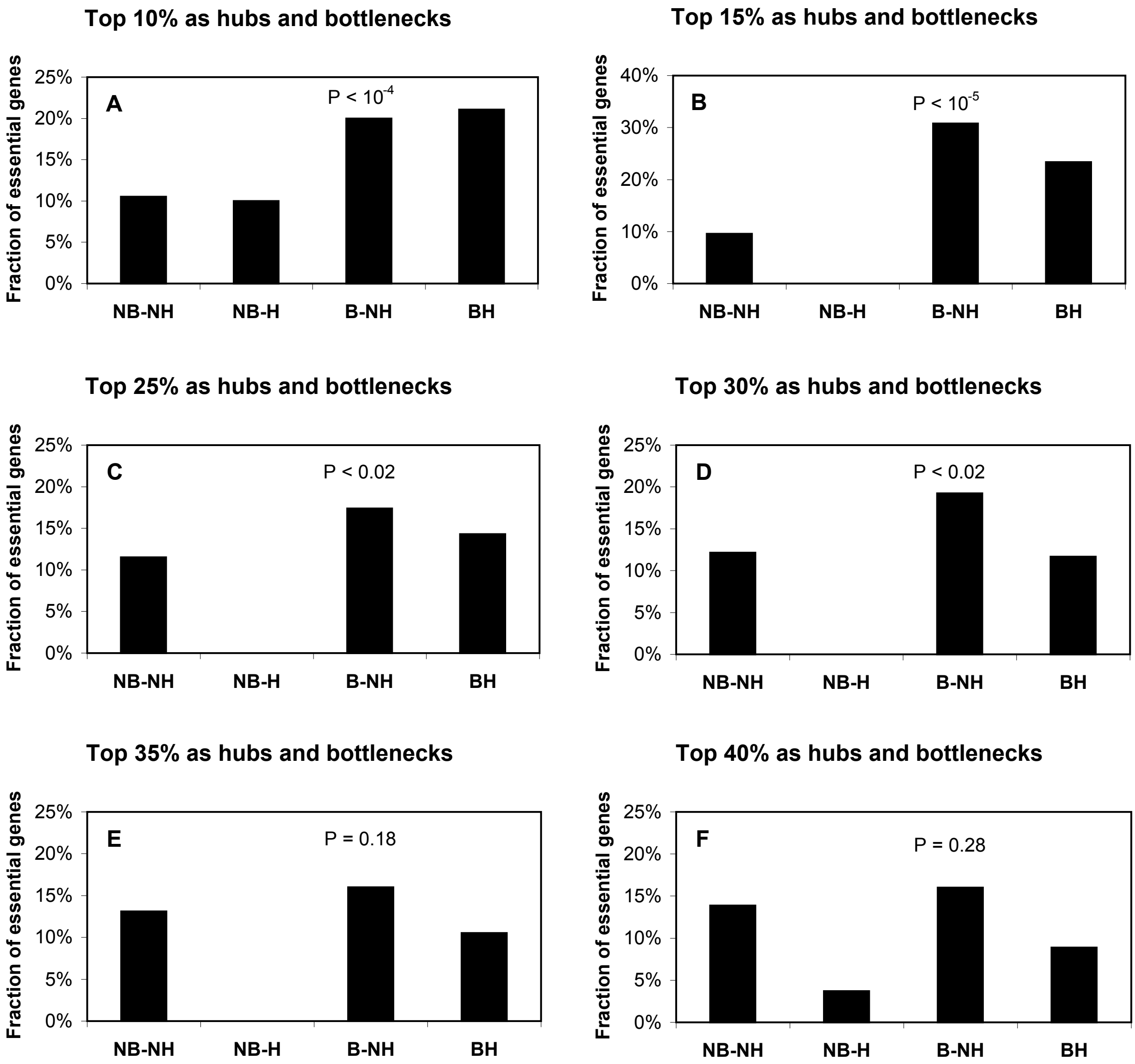

Top 10% as hubs and bottlenecks
A
P < 10-4
Top 15% as hubs and bottlenecks
B
P < 10-5
Top 25% as hubs and bottlenecks
C
P < 0.02
Top 30% as hubs and bottlenecks
D
P < 0.02
Top 35% as hubs and bottlenecks
E
P = 0.18
Top 40% as hubs and bottlenecks
F
P = 0.28
Fraction of essential genes
0%
5%
10%
15%
20%
25%
30%
40%
NB-NH
NB-H
B-NH
BH

SupFig5B
