# Supplementary material for: The Importance of Bottlenecks in Protein Networks: Correlation with Gene Essentiality and Expression Dynamics
Source: PLoS Comput Biol. 2007 Apr 20;3(4):e59. doi: 10.1371/journal.pcbi.0030059 (PMC1853125; doi:10.1371/journal.pcbi.0030059)
Supplement: Supplementary file 6 [file pcbi.0030059.sg006.pdf]

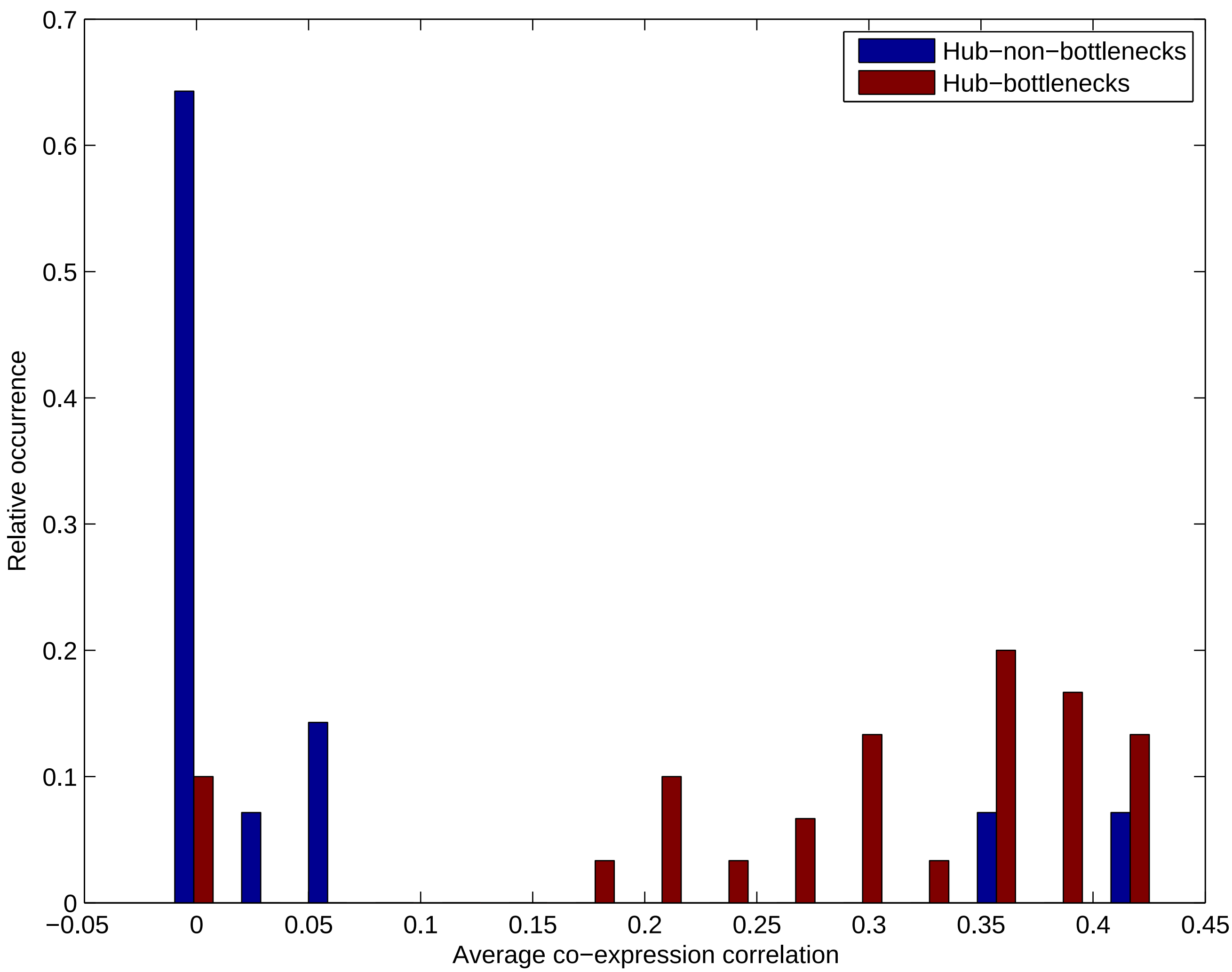
Hub−non−bottlenecks
Hub−bottlenecks
Relative occurrence
Average co−expression correlation
0
0.1
0.2
0.3
0.4
0.5
0.6
0.7
−0.05
0
0.05
0.1
0.15
0.2
0.25
0.3
0.35
0.4
0.45

SupFig6A

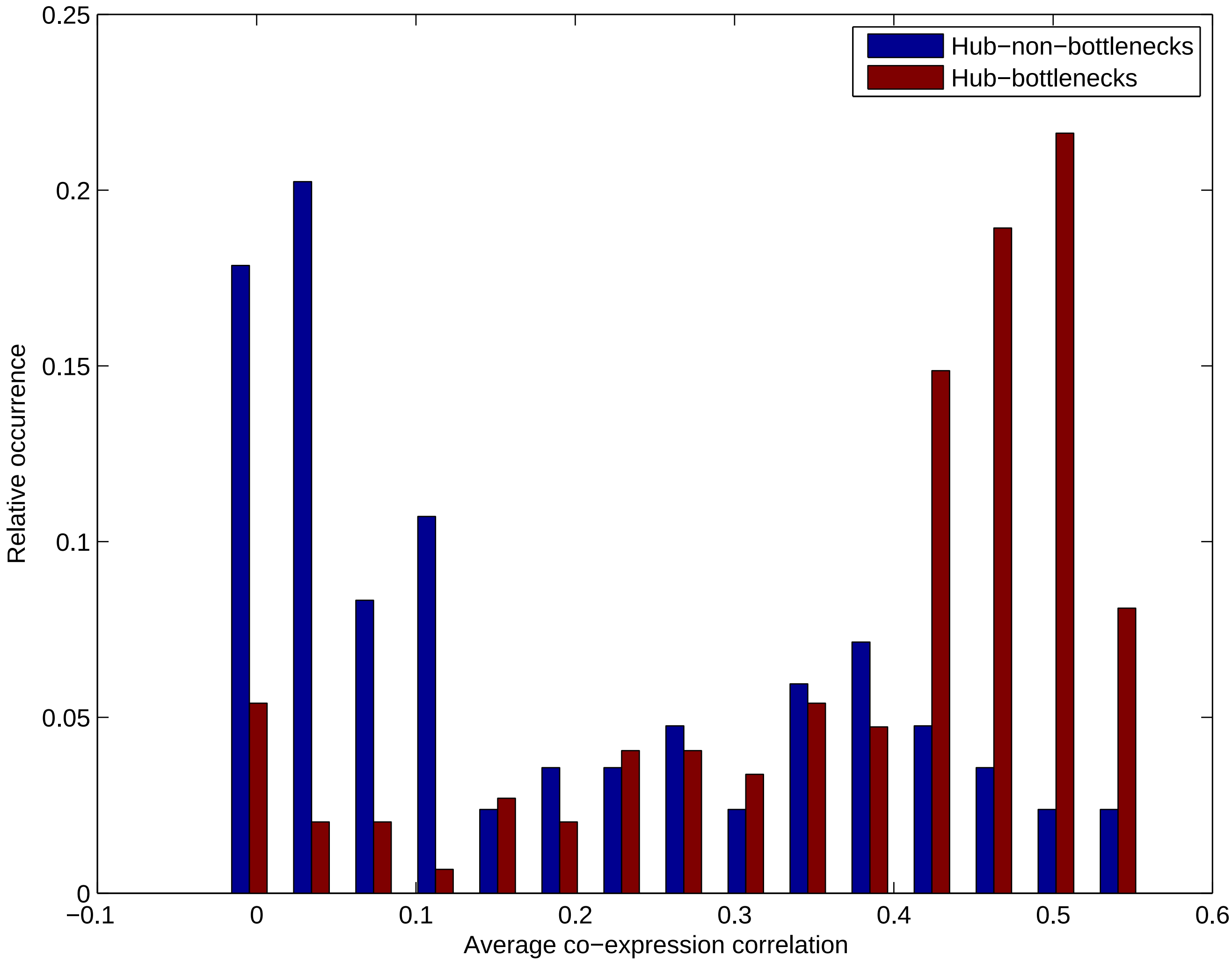
Hub−non−bottlenecks
Hub−bottlenecks
0.25
0.2
0.15
0.1
0.05
0
Relative occurrence
−0.1
0
0.1
0.2
0.3
0.4
0.5
0.6
Average co−expression correlation

SupFig6B

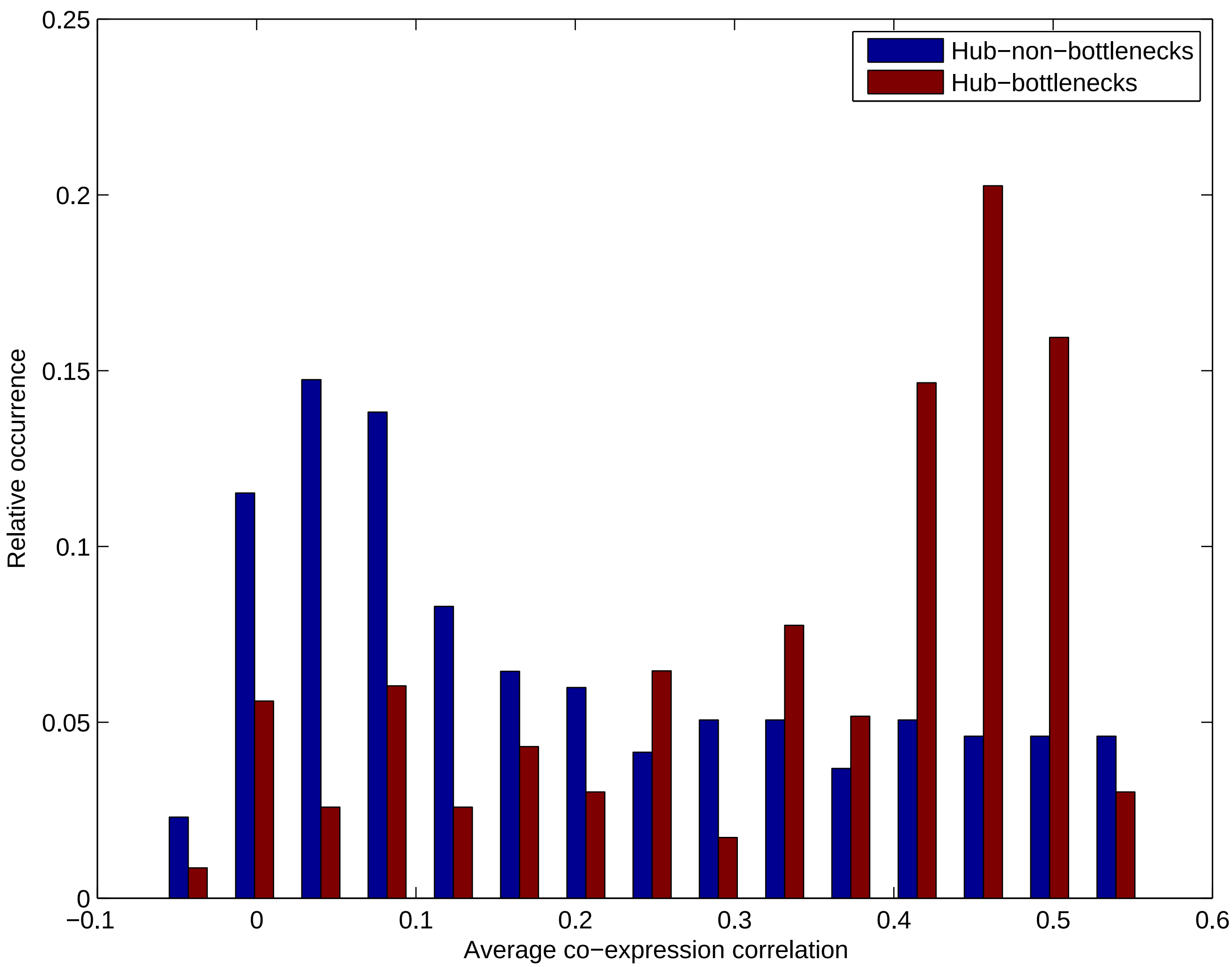

Hub−non−bottlenecks
Hub−bottlenecks
Relative occurrence
Average co−expression correlation
0.25
0.2
0.15
0.1
0.05
0
−0.1
0
0.1
0.2
0.3
0.4
0.5
0.6

SupFig6C

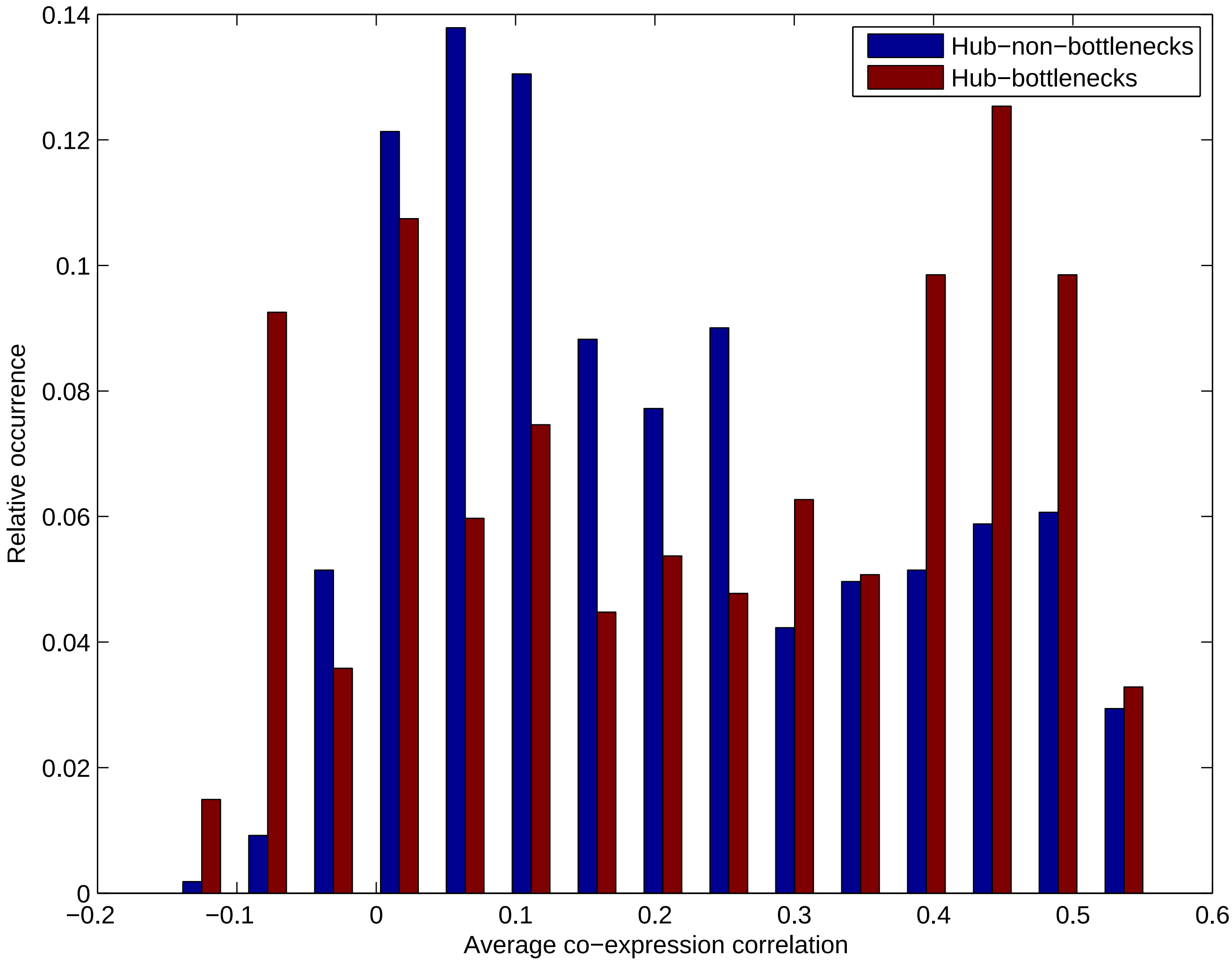
Hub−non−bottlenecks
Hub−bottlenecks
Relative occurrence
Average co−expression correlation
0.14
0.12
0.1
0.08
0.06
0.04
0.02
0
−0.2
−0.1
0
0.1
0.2
0.3
0.4
0.5
0.6

SupFig6D
