# Supplementary material for: The Importance of Bottlenecks in Protein Networks: Correlation with Gene Essentiality and Expression Dynamics
Source: PLoS Comput Biol. 2007 Apr 20;3(4):e59. doi: 10.1371/journal.pcbi.0030059 (PMC1853125; doi:10.1371/journal.pcbi.0030059)
Supplement: Supplementary file 7 [file pcbi.0030059.sg007.pdf]

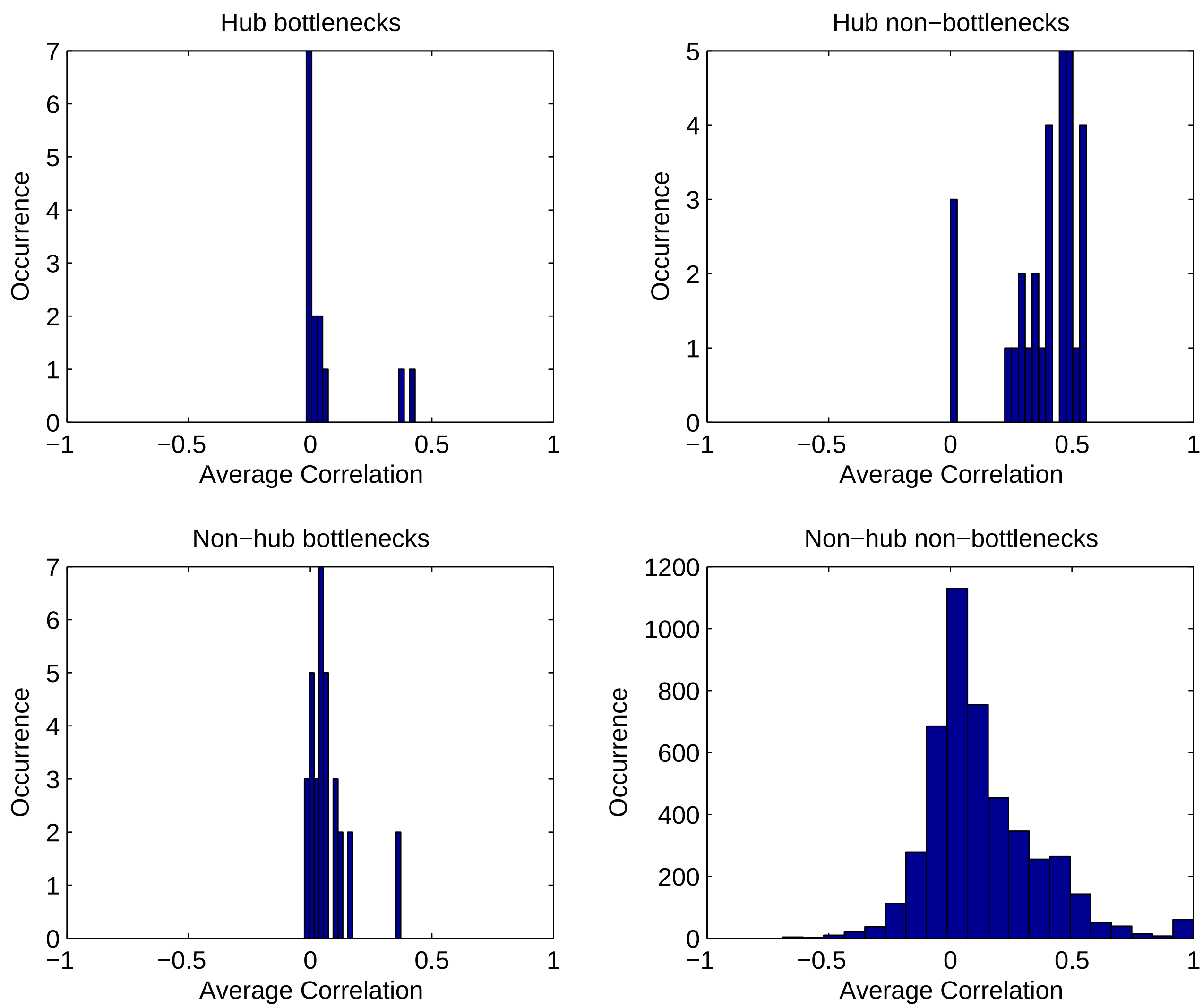

Hub bottlenecks
Hub non−bottlenecks
Non−hub bottlenecks
Non−hub non−bottlenecks
Occurrence
Average Correlation
−1
−0.5
0
0.5
1
0
1
2
3
4
5
6
7
200
400
600
800
1000
1200

SupFig7A

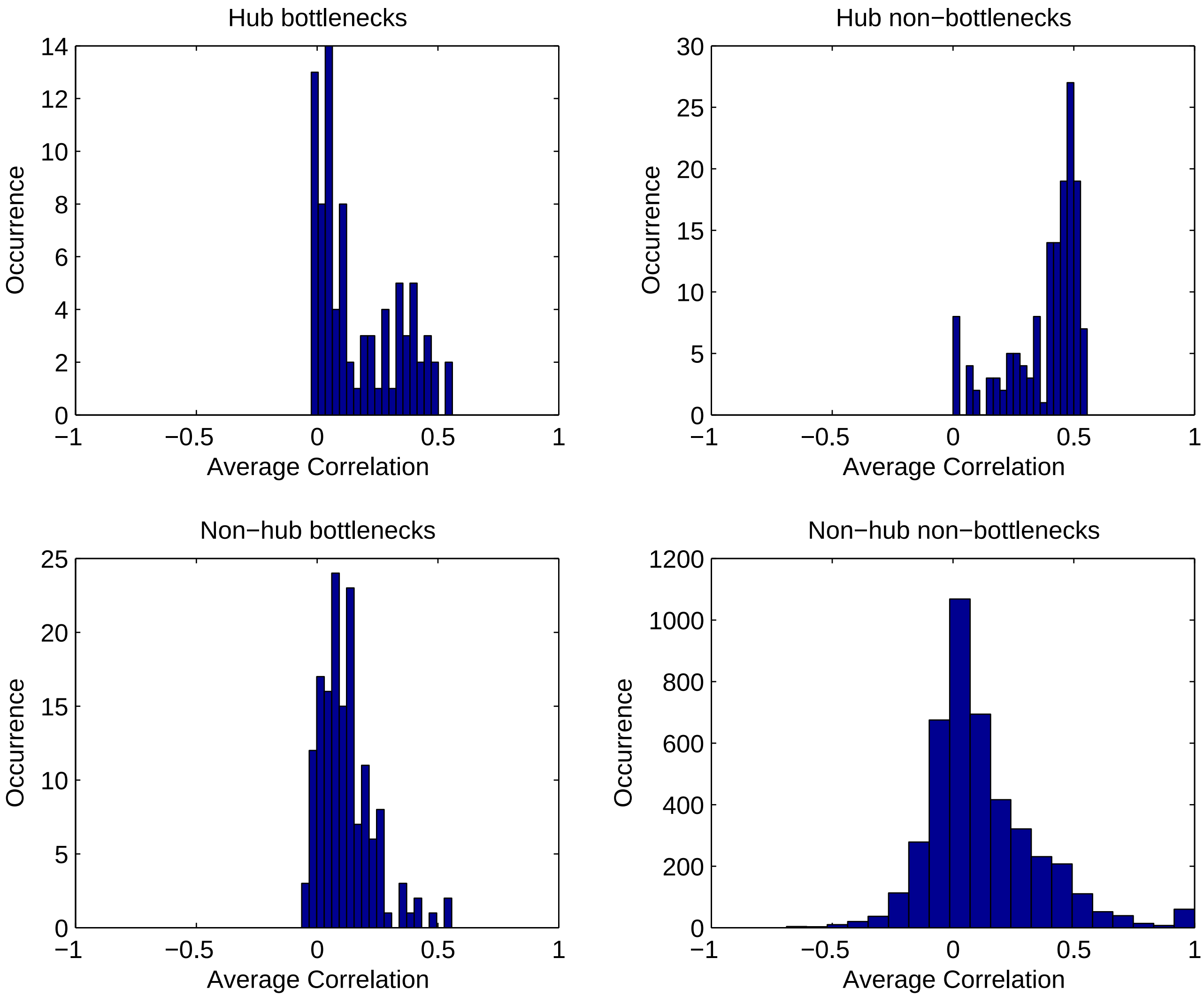

Hub bottlenecks
Hub non−bottlenecks
Non−hub bottlenecks
Non−hub non−bottlenecks
Occurrence
Average Correlation

SupFig7B

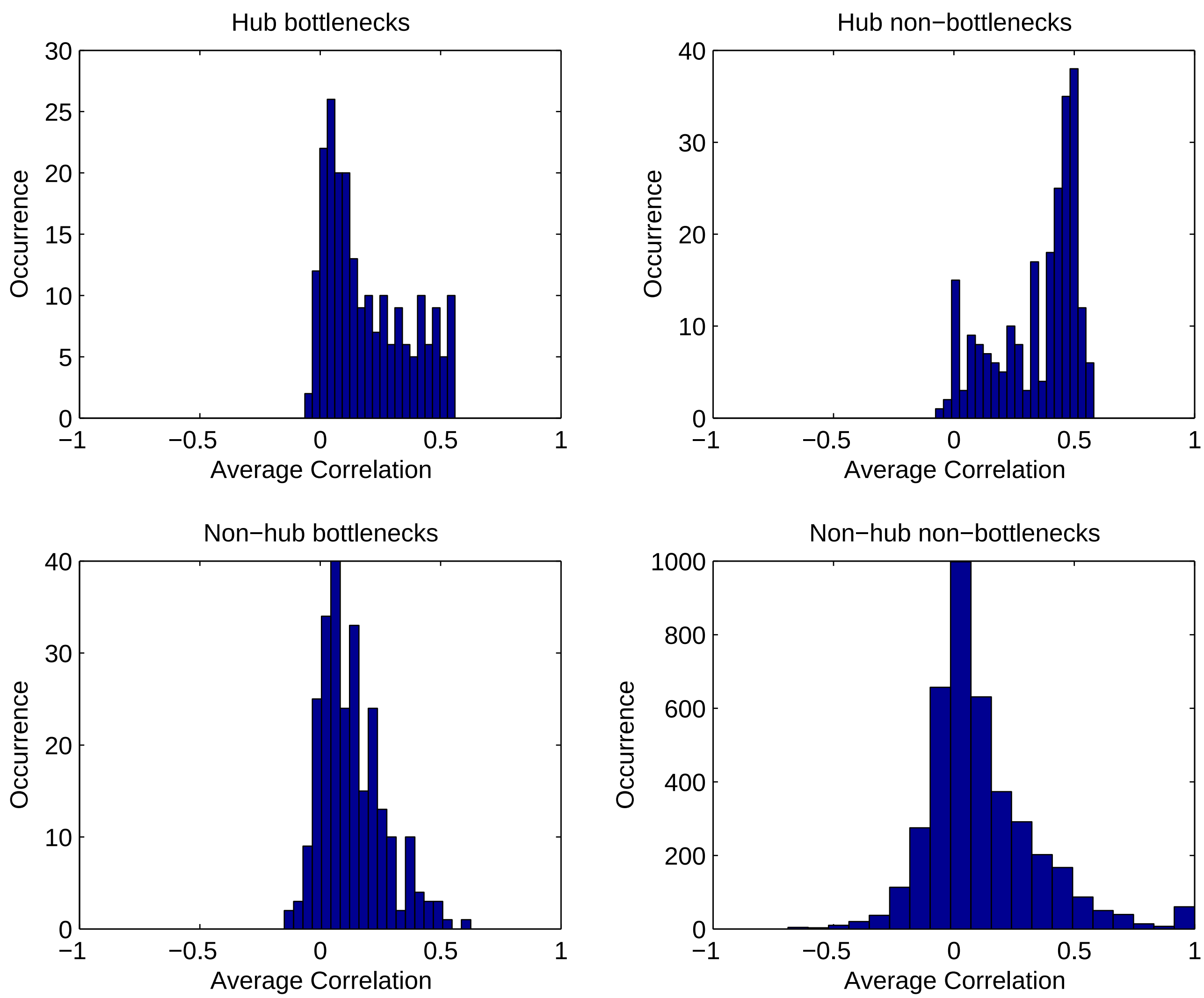
Hub bottlenecks
Hub non−bottlenecks
Non−hub bottlenecks
Non−hub non−bottlenecks
Occurrence
Average Correlation

SupFig7C

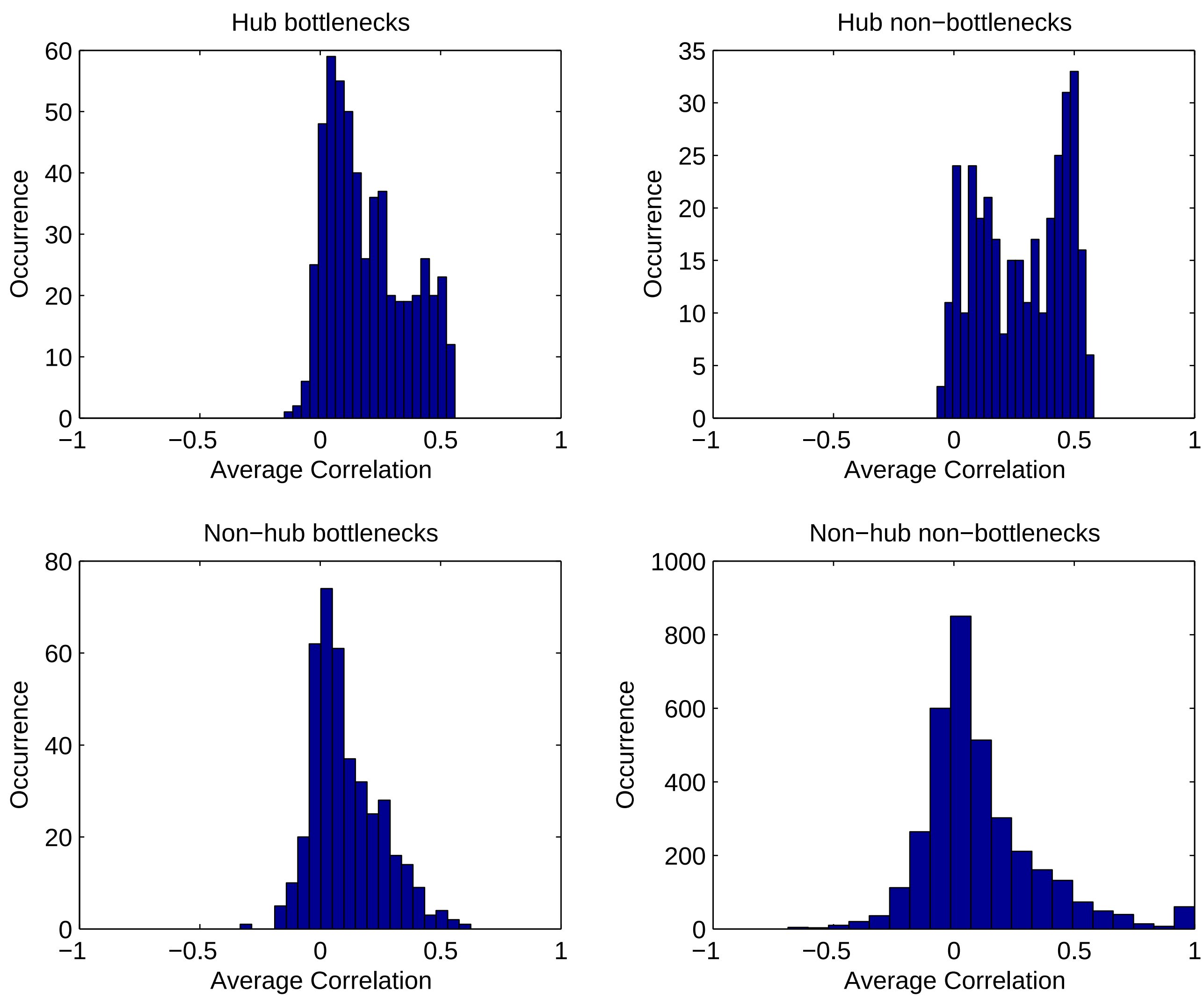
Hub bottlenecks
Hub non−bottlenecks
Non−hub bottlenecks
Non−hub non−bottlenecks
Occurrence
Average Correlation
0
10
20
30
40
50
60
5
15
25
35
80
200
400
600
800
1000
−1
−0.5
0.5
1

SupFig7D
