# Supplementary material for: The Importance of Bottlenecks in Protein Networks: Correlation with Gene Essentiality and Expression Dynamics
Source: PLoS Comput Biol. 2007 Apr 20;3(4):e59. doi: 10.1371/journal.pcbi.0030059 (PMC1853125; doi:10.1371/journal.pcbi.0030059)
Supplement: Supplementary file 8 [file pcbi.0030059.st001.doc]

# Supplementary Table 1. Number of proteins in each of the four categories in both interaction and regulatory networks.
