# Supplementary material for: The Importance of Bottlenecks in Protein Networks: Correlation with Gene Essentiality and Expression Dynamics
Source: PLoS Comput Biol. 2007 Apr 20;3(4):e59. doi: 10.1371/journal.pcbi.0030059 (PMC1853125; doi:10.1371/journal.pcbi.0030059)
Supplement: Supplementary file 9 [file pcbi.0030059.st002.doc]

# Supplementary Table 2. Essentiality of different categories of proteins in phosphorylation, metabolic and genetic networks.

|  | **Non-hub-non-bottlenecks** | **Hub-non-bottlenecks** | **Non-hub-bottlenecks** | **Hub-bottlenecks** |
| --- | --- | --- | --- | --- |
| **Genetic Network** | 21.37% | 14.08% | 23.94% | 13.61% |
| **Metabolic Network** | 21.15% | 19.80% | 23.76% | 7.69% |
| **Phosphorylation Network** | 17.57% | 20.77% | 19.13% | 7.41% |
