# Supplementary material for: The Importance of Bottlenecks in Protein Networks: Correlation with Gene Essentiality and Expression Dynamics
Source: PLoS Comput Biol. 2007 Apr 20;3(4):e59. doi: 10.1371/journal.pcbi.0030059 (PMC1853125; doi:10.1371/journal.pcbi.0030059)
Supplement: Supplementary file 10 [file pcbi.0030059.st003.doc]

# Supplementary Table 3. Occurrence of different biological process annotations from the Gene Ontology annotation scheme in the four different topological categories.

| **Hub-bottlenecks** |  |  |  |
| --- | --- | --- | --- |
| Biological Process | Occurrence % | Molecular function | Occurrence % |
| protein biosynthesis | 10.64% | molecular_function unknown | 14.31% |
| ubiquitin-dependent protein catabolism | 6.06% | structural constituent of ribosome | 10.09% |
| nuclear mRNA splicing, via spliceosome | 5.50% | protein binding | 5.32% |
| biological_process unknown | 3.85% | RNA binding | 3.30% |
| protein amino acid phosphorylation | 3.12% | pre-mRNA splicing factor activity | 3.12% |
| **Hub-non-bottlenecks** |  |  |  |
| Biological Process | Occurrence % | Molecular function | Occurrence % |
| protein biosynthesis | 34.43% | structural constituent of ribosome | 34.13% |
| rRNA processing | 3.59% | molecular_function unknown | 8.68% |
| ubiquitin-dependent protein catabolism | 3.59% | protein binding | 3.89% |
| 35S primary transcript processing | 3.29% | general RNA polymerase II transcription factor activity | 2.69% |
| nuclear mRNA splicing, via spliceosome | 3.29% | snoRNA binding | 2.40% |
| **Non-hub-bottlenecks** |  |  |  |
| Biological Process | Occurrence % | Molecular function | Occurrence % |
| biological_process unknown | 15.63% | molecular_function unknown | 26.05% |
| protein amino acid phosphorylation | 2.23% | protein binding | 3.97% |
| regulation of transcription from Pol II promoter | 2.23% | unfolded protein binding | 1.99% |
| protein biosynthesis | 1.99% | protein transporter activity | 1.99% |
| response to stress | 1.74% | transcription factor activity | 1.99% |
| **Non-hub-non-bottlenecks** |  |  |  |
| Biological Process | Occurrence % | Molecular function | Occurrence % |
| biological_process unknown | 25.25% | molecular_function unknown | 35.08% |
| cell wall organization and biogenesis | 1.70% | protein binding | 2.80% |
| protein amino acid phosphorylation | 1.65% | DNA binding | 1.21% |
| protein biosynthesis | 1.50% | protein kinase activity | 1.01% |
| DNA repair | 1.21% | transcription factor activity | 0.84% |
